# Evaluation and mitigation of deformable image registration uncertainties for MRI‐guided adaptive radiotherapy

**DOI:** 10.1002/acm2.14358

**Published:** 2024-04-18

**Authors:** Hualiang Zhong, Kristofer K. Kainz, Eric S. Paulson

**Affiliations:** ^1^ Department of Radiation Oncology Medical College of Wisconsin Milwaukee Wisconsin USA

**Keywords:** adaptive radiotherapy, deformable image registration, evaluation metrics, magnetic resonance image, uncertainty reduction

## Abstract

**Purpose:**

We evaluate the performance of a deformable image registration (DIR) software package in registering abdominal magnetic resonance images (MRIs) and then develop a mechanical modeling method to mitigate detected DIR uncertainties.

**Materials and Methods:**

Three evaluation metrics, namely mean displacement to agreement (MDA), DICE similarity coefficient (DSC), and standard deviation of Jacobian determinants (STD‐JD), are used to assess the multi‐modality (MM), contour‐consistency (CC), and image‐intensity (II)‐based DIR algorithms in the MIM software package, as well as an in‐house developed, contour matching‐based finite element method (CM‐FEM). Furthermore, we develop a hybrid FEM registration technique to modify the displacement vector field of each MIM registration. The MIM and FEM registrations were evaluated on MRIs obtained from 10 abdominal cancer patients. One‐tailed Wilcoxon‐Mann‐Whitney (WMW) tests were conducted to compare the MIM registrations with their FEM modifications.

**Results:**

For the registrations performed with the MIM‐CC, MIM‐MM, MIM‐II, and CM‐FEM algorithms, their average MDAs are 0.62 ± 0.27, 2.39 ± 1.30, 3.07 ± 2.42, 1.04 ± 0.72 mm, and average DSCs are 0.94 ± 0.03, 0.80 ± 0.12, 0.77 ± 0.15, 0.90 ± 0.11, respectively. The *p*‐values of the WMW tests between the MIM registrations and their FEM modifications are less than 0.0084 for STD‐JDs and greater than 0.87 for MDA and DSC.

**Conclusions:**

Among the three MIM DIR algorithms, MIM‐CC shows the smallest errors in terms of MDA and DSC but exhibits significant Jacobian uncertainties in the interior regions of abdominal organs. The hybrid FEM technique effectively mitigates the Jacobian uncertainties in these regions.

## 1 INTRODUCTION

Magnetic resonance (MR) linear accelerators enable the routine acquisition of daily MR images for assessing dosimetry changes in targets and organs at risk (OARs), which could facilitate subsequent plan adaptation if necessary.^1^ Various techniques have been developed to streamline the adaptation process, including auto‐segmentation,^2^ real‐time MR imaging,^3^ and deformable dose accumulation (DDA).^4^ DDA relies on deformable image registration (DIR) techniques to derive displacement vector fields that can be used to transfer doses from daily MR images to a consensus image for dose comparison or assessment.^5^ Several factors could compromise MRI registrations. For instance, physiological phenomena such as variations in water content can alter the anatomical appearance of daily MRIs; radiation treatment can induce anatomical changes like lung tissue inflammation, tumor shedding, or non‐elastic regression.^6^ These variables can mislead image‐based DIR algorithms that use mutual information, sum of squared differences, or feature consistencies as their objective functions. Consequently, this compromises the quality of downstream applications, including DDA and treatment response assessment.^7^

Several metrics have been recommended for evaluating DIR performances, including mean distance to agreement (MDA) and DICE similarity coefficient (DSC) for surface comparison, as well as Jacobian determinant (JD) and inverse consistency error (ICE) for voxel‐wise assessment.^8^ These evaluation metrics, coupled with their clinical tolerances, provide valuable guidance for clinical practice.^4^ However, when registration errors exceed acceptable clinical thresholds, rectifying these errors becomes paramount. In contrast to image‐based registration algorithms, which are susceptible to adverse image characteristics such as imaging artifacts and low image contrasts,^9^ mechanical modeling techniques, being independent of such characteristics, present a promising avenue for mitigating errors in image‐based registrations.^10^

The purpose of this study is twofold, evaluating the performance of DIR algorithms implemented in a commercial software package and developing a mechanical modeling method to mitigate identified DIR uncertainties. Specifically, we first evaluate the performance of four DIR algorithms, and then develop a finite element method (FEM)‐based technique to reduce identified registration errors. The developed technique has two approaches to configure its boundary conditions, depending on the availability of contours on the images being registered. We will demonstrate the feasibility and efficiency of applying the developed technique to MR images acquired from MR‐Linac patients.

## 2 METHODS

### 2.1 Deformable image registration methods

**FIGURE 1:**
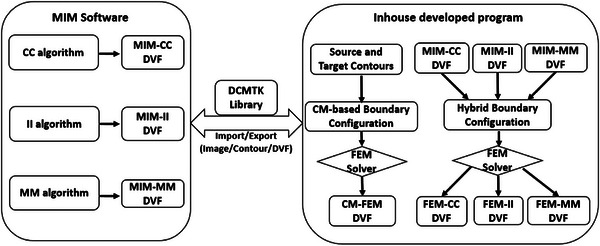
Diagram of the in‐house developed FEM program interfacing with the MIM software.

In this study we first evaluated three DIR algorithms (MIM‐CC, MIM‐II, and MIM‐MM) in MIM (MIM Software Inc., Cleveland, OH), and then developed a hybrid FEM program to modify the outputs from each of these algorithms. Additionally, we introduced a contour matching technique for direct FEM registrations. The developed program communicates with the MIM software through DICOM import and export, as illustrated in Figure 1.

#### 2.1.1 DIR methods in MIM software

The MIM software (version 7.06) includes three DIR methods (MIM‐II, MIM‐MM, MIM‐CC) that employ the image‐intensity, multi‐modality and contour‐consistency‐based objective functions as their similarity metrics, respectively. MIM‐CC minimizes the difference of contour surfaces between the two registered images using a modified gradient descent method, and MIM‐MM maximizes the correspondence of high dimensional feature descriptors at each image voxel using the Gauss‐Newton‐based optimization method. MIM‐II is an image intensity‐based, free‐form deformable registration algorithm, where the squared differences of normalized intensities are summed as a similarity metric and minimized using the modified gradient descent method.^11^ In the commercial software, users do not have access to any optimization parameters. However, when contours are used for guidance in MIM‐CC registrations, users can specify how many and what contours to include. In this study, we included 20 OARs, as specified in the supplementary material, for DIR evaluation, and their contours were used to guide MIM‐CC registrations.

#### 2.1.2 Contour matching‐based FEM registration

In this study, we develop a contour matching‐based FEM registration technique as described below. When OARs are contoured on both reference and moving images, displacements at contoured points in the reference image can be derived. Specifically, for each contour *R* on the reference image, we first identify its counterpart (*M*) on the moving image and then perform a rigid translation by moving the center of M to the center of R. The translated contour is denoted by *M*_T_. We define an objective function as follows:

(1) $$\Omega \;\left(R,\;M_{T}\right)=\sum_{i}\min_{j\in L_{i}}\left\{G\left(r_{i},m_{t,j}\right)+\lambda N\left(r_{i},m_{t,j}\right)\right\}\;$$

Here, *G*(*r*_i_, *m*_t,j_) represents the geometric distance between the contour point *r*_i_ and *m*_t,j_, and *N*(*r*_i_, *m*_t,j_) denotes the local similarity between *r*_i_ and *m*_t,j_. *L*_i_ is a set of moving points located within a given distance of *r*_i_. The parameter λ can be adjusted according to the deformation of individual OARs, with its default value set to 1.0. *N*(*r*, *m*) is defined as

(2) $$N\;\left(r,\;m\right)=\sum_{k\in B_{r}}g_{k}\left(I_{r,k}-I_{m,k}\right)\;$$

where *B*_r_ represents the neighborhood of *r*. *I*_r,k_ is 1 if voxel *k* in the reference image contains a point of the contour *R*; otherwise, *I*_r,k_ is 0. The same definition applies to *I*_m,k_ for contour *M*. *g*_k_ represents the corresponding Gaussian function defined between *r* and *k*. The displacement vector at each contour point is derived by minimizing $\Omega (R,\;M_{T})$. Repeating the above procedure for each of these OARs, we can derive displacements at all contour points. These displacements will be used as boundary constraints in a mechanical model to calculate a displacement vector field (DVF) on the reference image. This approach is named as a contour matching‐based finite element method (CM‐FEM).

#### 2.1.3 Hybrid FEM registration

We also develop a hybrid registration method for each DIR algorithm in MIM. Different from the above CM‐FEM method, we perform a MIM registration to derive boundary constraints for mechanical modeling. Specifically, for the MIM registration, we identify boundary voxels from contours on its reference image and derive displacements at these voxels from its DVF. We scale a cubic tetrahedral mesh consisting of 131 614 nodes and 747 384 tetrahedron to cover all the boundary voxels. Then, we establish a lookup table between the indices of tetrahedral nodes and the image voxels covered by the mesh. Consequently, given a boundary voxel, its closest tetrahedral node can be identified. Displacements at these nodes can be derived from the MIM registration and used as boundary constraints in a mechanical model.

A volumetric interpolation is then employed to convert the model‐generated displacements into a new DVF on the reference image. With this approach, the hybrid methods FEM‐II, FEM‐MM, and FEM‐CC are developed in correspondence to MIM‐II, MIM‐MM, and MIM‐CC, respectively. It is worth noting that different preliminary registrations can be performed for each organ, with resultant displacements integrated to generate a mixed configuration file for the subsequent mechanical modeling.

### 2.2 FEM‐based mechanical modeling

An in‐house developed finite element program was adapted to calculate tissue deformation for organs contoured on a reference image.^12^ Specifically, we configured the Young's modulus (*E*) and Poisson's ratio (*v*) of each element in a scaled tetrahedral mesh according to the element's location, and then converted the governing equation of elasticity to a set of linear algebraic equations^12^:

(3) $$K*\;\left[\begin{array}{c} d_{1}\\ \text{…}\\ d_{k}\\ \begin{array}{c} d_{k+1}\\ \text{…}\\ d_{n} \end{array} \end{array}\right]=\left[\begin{array}{c} \begin{array}{c} F_{1}-\sum_{i}g_{1,i}d_{i}\\ \text{…}\\ F_{k}-\sum_{i}g_{k,i}d_{i} \end{array}\\ \begin{array}{c} -\sum_{i}g_{k+1,i}d_{i}\\ \text{…}\\ -\sum_{i}g_{n,i}d_{i} \end{array} \end{array}\right]\;.$$

Here, *K* represents the assembled global stiffness matrix, and *F_1_,…,F_k_* represent the external forces acting on driving nodes, with *d_1_,…,d_k_* representing displacement constraints pre‐assigned to these nodes. The displacements of non‐driving nodes are denoted by *d_k+1_,…,d_n_*. The material parameters used in the matrix *K* are consistent with the reported data.^13^ Specifically, Young's moduli were set to 1 kPa for the lung, 10 kPa for soft tissue, and 1 MPa for bones, while the Poisson ratios were set to 0.38 for the lung, 0.45 for soft issue, and 0.49 for bones.

The displacement *d_1_,…,d_k_* can be either interpolated from the DVF of an MIM registration or generated by the contour matching technique. After modeling tissue deformation, the derived displacements at each node are assigned to image voxels using a volumetric interpolation method.^13^ The interpolated displacement vectors are combined with those pre‐assigned to voxels outside the mesh to generate a modified DVF. The contour matching and mechanical modeling techniques are implemented with C++ on a Linux computer. With the DCMTK toolkit,^14^ modified DVFs are saved in DICOM files, which can be imported directly into the MIM software for comparison with other DIR algorithms.

### 2.3 Images and DIR evaluation metrics

#### 2.3.1 MR images and data acquisition

The DIR algorithms were evaluated on MR images acquired from 10 abdominal cancer patients aged 18 or older, who underwent treatment with adapt‐to‐shape (ATS) on an Elekta MR‐Linac machine. For each patient, the number of treatment fractions requiring ATS may vary, depending on anatomical deformation or changes observed from daily images.^15^ A 3D Vane sequence‐based, respiratory‐correlated imaging protocol was used to acquire T1‐weighted 4D images and an average 3D image of in‐plane resolution 1.6 mm and slice thickness 2.38 mm.^16^ The average 3D image was reconstructed with fully acquired data and used for treatment planning in ATS. On the average image, critical OARs close to treatment target were fully contoured following a published protocol,^16^ and were reviewed by an experienced physician for plan adaptation.

#### 2.3.2 Deformable image registrations

For each patient, a deformable registration was performed from the second ATS fraction to the first ATS fraction, using the algorithms MIM‐II, MIM‐MM, MIM‐CC, and CM‐FEM, respectively. For each MIM registration, its DVF was modified with the hybrid FEM technique. Resultant DVFs were evaluated within 1–3 critical OARs that are fully contoured in ATS for each patient. These OARs, as specified in the supplementary material, are near treatment targets and generally experience high dose gradients.

As an example of utilizing the hybrid FEM technique, dose transfer was performed for one patient, using DVFs generated by MIM‐CC and the hybrid FEM technique, respectively. The “Transfer Dose” function in MIM was used to transfer dose from the second ATS fraction to the first ATS fraction. The transferred doses were compared using DVHs and illustrated with color washes.

#### 2.3.3 DIR evaluation metrics

MIM and FEM registrations performed in this study were evaluated using the metrics DSC, MDA, and JD, with their means and standard deviations calculated for individual organs and illustrated using boxplots. One‐tailed Wilcoxon‐Mann‐Whitney (WMW) tests^17^ were programmed with Excel for Microsoft 365 (Microsoft Corporation, Redmond, Washington) to determine the statistical significance of FEM improvements. The thresholds of 3 mm in MDA and 0.8 in DSC were used to evaluate DIRs at organ boundaries,^4^ and the range from 0.8 to 1.2 for JD was utilized for evaluation of DIRs in soft tissue regions.^8^ It is important to note that JD values could fall outside that range if an organ undergoes significant volume changes. In this study, we utilize the standard deviation of JD (STD‐JD) to evaluate DIR performance in individual OARs.^18^, ^19^ It should be mentioned that these metrics and thresholds are employed in this study mainly for relative comparisons between different DIR algorithms and not as acceptance criteria for dose accumulation. In the latter case, additional factors such as organ volume variations and dose gradients should be considered.^20^, ^21^

## 3 RESULTS

### 3.1 Evaluation of four DIR algorithms

**FIGURE 2:**
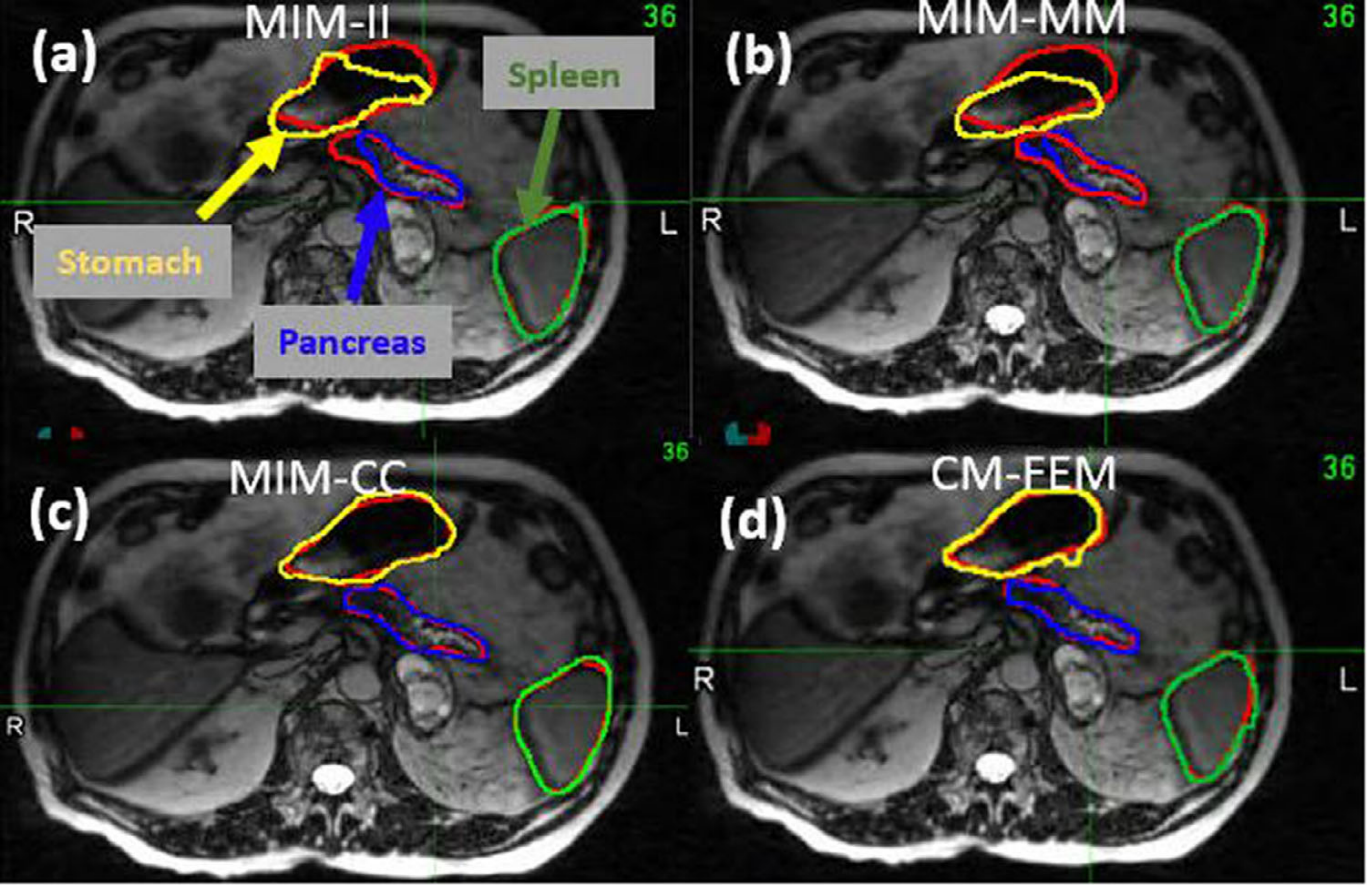
The reference image of patient 1: the target contours (red) of pancreas, spleen and stomach overlapped with their warped contours by (a) MIM‐II, (b) MIM‐MM, (c) MIM‐CC, and (d) CM‐FEM, respectively.

The four DIR algorithms (MIM‐II, MIM‐MM, MIM‐CC, and CM‐FEM) were applied to MR images acquired from 10 MR‐Linac patients. The performance of these algorithms was evaluated on individual OARs. As illustrated in Figure 2a–d, the warped pancreas, spleen, and stomach contours were colored in blue, green, and yellow, respectively, while their target contours were colored in red. The two image‐based algorithms (MIM‐II and MIM‐MM) exhibited inferior performance compared to MIM‐CC and CM‐FEM in the contour comparisons. The differences in performances could be partly attributed to the impact of water variations within the stomach between the two registered images. A similar phenomenon was observed in the small bowel, where filling changes significantly impacted the MIM‐II registration, resulting in the warped contour for the left kidney being off by as far as 11 mm in MDA (see Figure S1). Material changes in the stomach and small bowel not only compromise the performance of an image‐based registration within these structures but also affect their surrounding areas.

**FIGURE 3:**
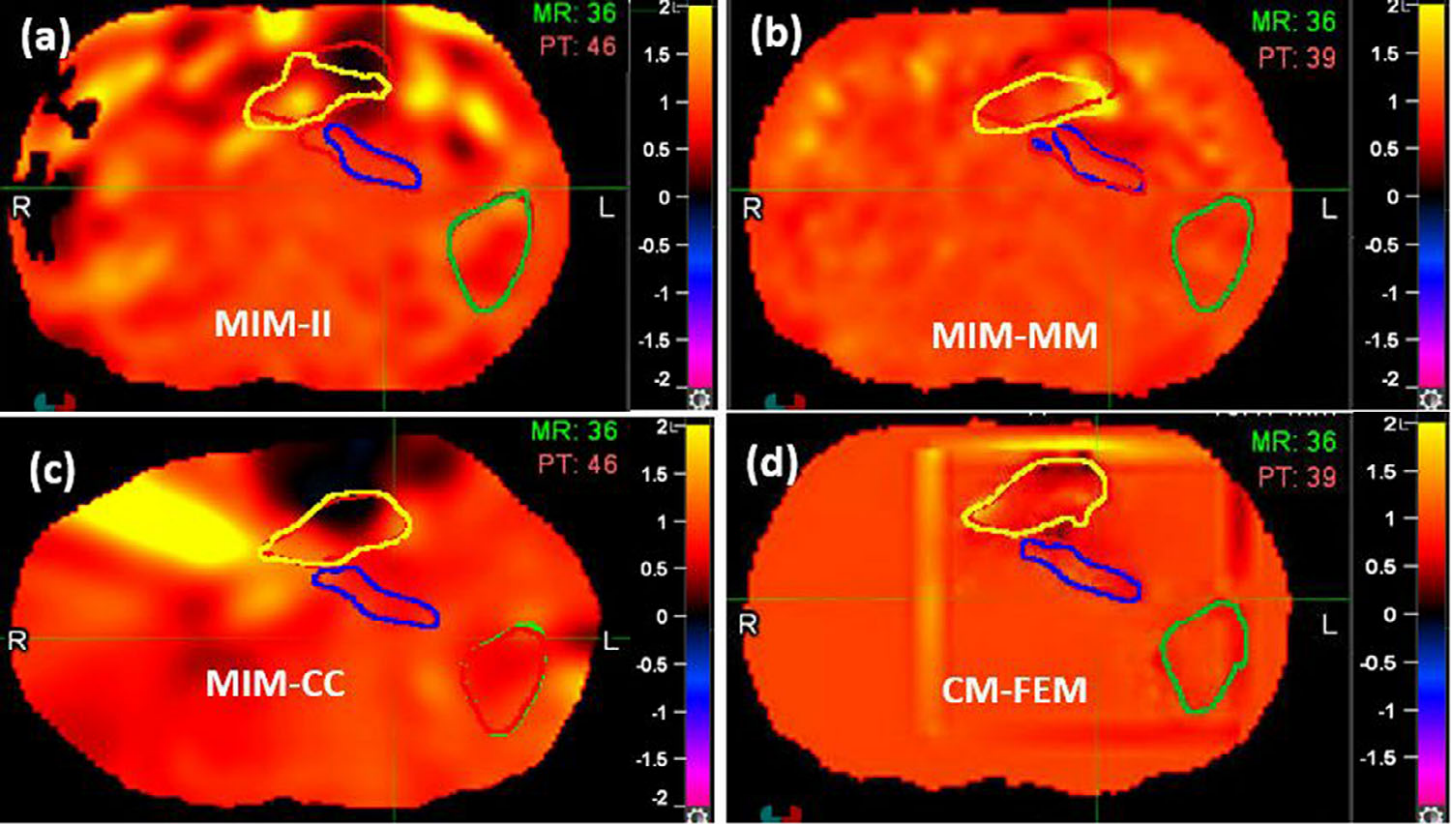
JD maps of the four registrations calculated for patient 1: (a) MIM‐II, (b) MIM‐MM, (c) MM‐CC, and (d) CM‐FEM.

For the four DIRs shown in Figure 2, their JD distributions were illustrated in Figure 3. In comparison to CM‐FEM, MIM‐II, MIM‐MM, and MIM‐CC exhibit noticeable hot or cold spots, indicating large standard deviations in their JD distributions. Among the three MIM DIR algorithms, MIM‐CC demonstrates the best performance at organ boundaries but produces the largest uncertainty in the interior of the stomach.

The four DIR algorithms were evaluated on the 20 OARs. The averaged MDAs were 3.07 ± 2.42, 2.39 ± 1.30, 0.62 ± 0.27, and 1.04 ± 0.72 mm for MIM‐II, MIM‐MM, MIM‐CC, and CM‐FEM, respectively, and the averaged DSCs were 0.77 ± 0.15, 0.8 ± 0.12, 0.94 ± 0.03, and 0.90 ± 0.11. DIR uncertainties in these organs have an average STD‐JD of 0.44 ± 0.39, 0.24 ± 0.14, 0.33 ± 0.29, and 0.21 ± 0.09 for the four algorithms, respectively. Boxplots for these metrics are reported in the Figures S2–S4.

### 3.2 Evaluation of the hybrid FEM algorithm

**FIGURE 4:**
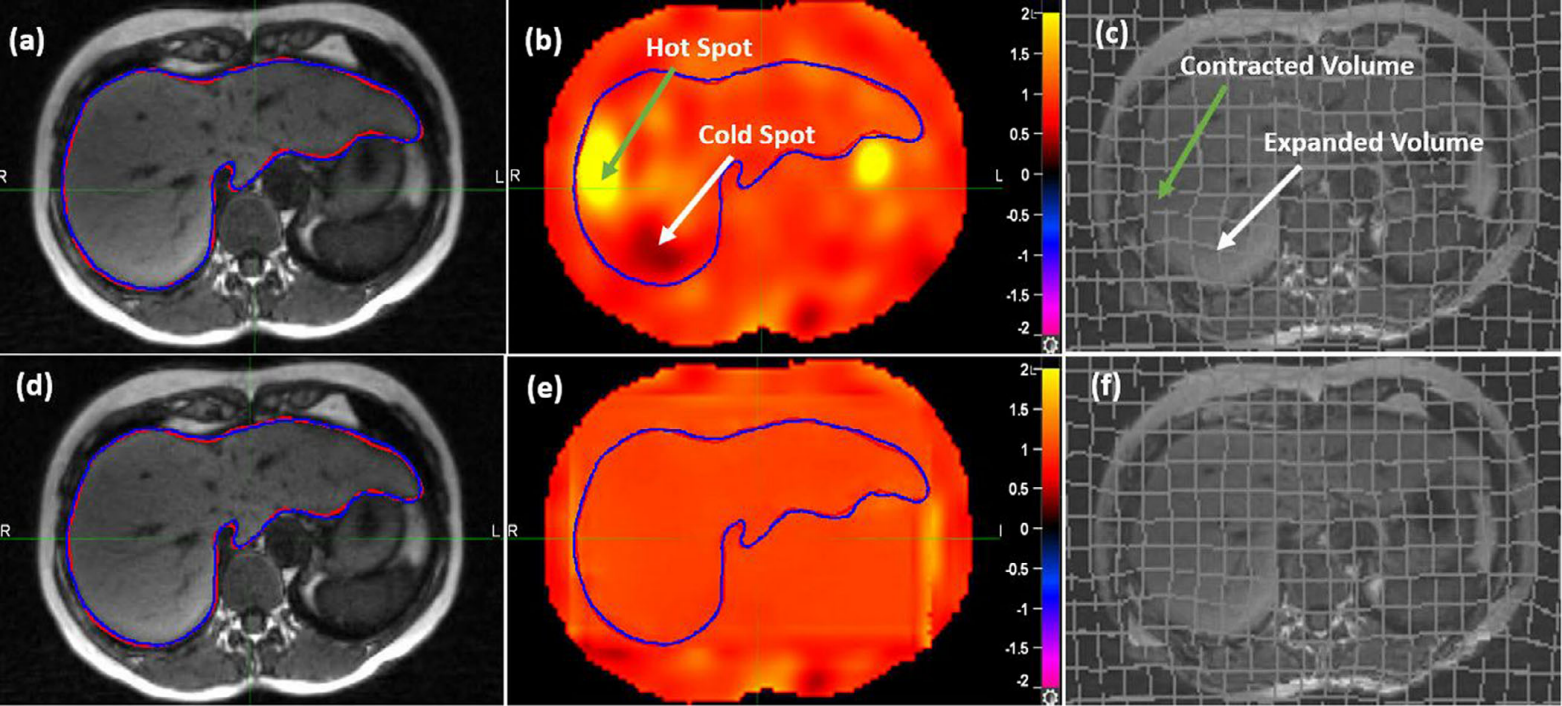
The transversal cut of the reference image for patient 2: (a) the reference liver contour compared with the MIM‐II warped contour, (b) the JD map of the MIM‐II registration, (c) the grided source image deformed by the MIM‐II generated DVF, (d–f) the corresponding slices for the FEM‐II registration.

As shown in Figure 4a, an MIM‐II registration was performed from a treatment image to the reference image for a patient with hepatocellular carcinoma, where the liver contour (blue) warped by the registration highly overlaps with its target contour (red). However, the JD map of the registration (Figure 4b) exhibits large hot and cold spots. To interpret the hot and cold spots, the source image was marked with grids at 1 cm intervals and then warped using a backward mapping method.^22^ The warped grids manifest volume contraction and expansion at the locations of the hot and cold spots (Figure 4c), respectively. It is essential to note that the liver is nearly incompressible, and a sub‐volume within the liver should not experience expansion or contraction during deformation.^23^ Consequently, the hot and cold spots in Figure 4b indicate registration errors. To rectify these errors, the FEM modeling technique was applied to the DVF of the MIM‐II registration. After the FEM modification, the warped contours remain consistent with the reference contours (Figure 4d), but the hot spots and irregular deformations within the liver have been successfully rectified (Figure 4e,f).

**FIGURE 5:**
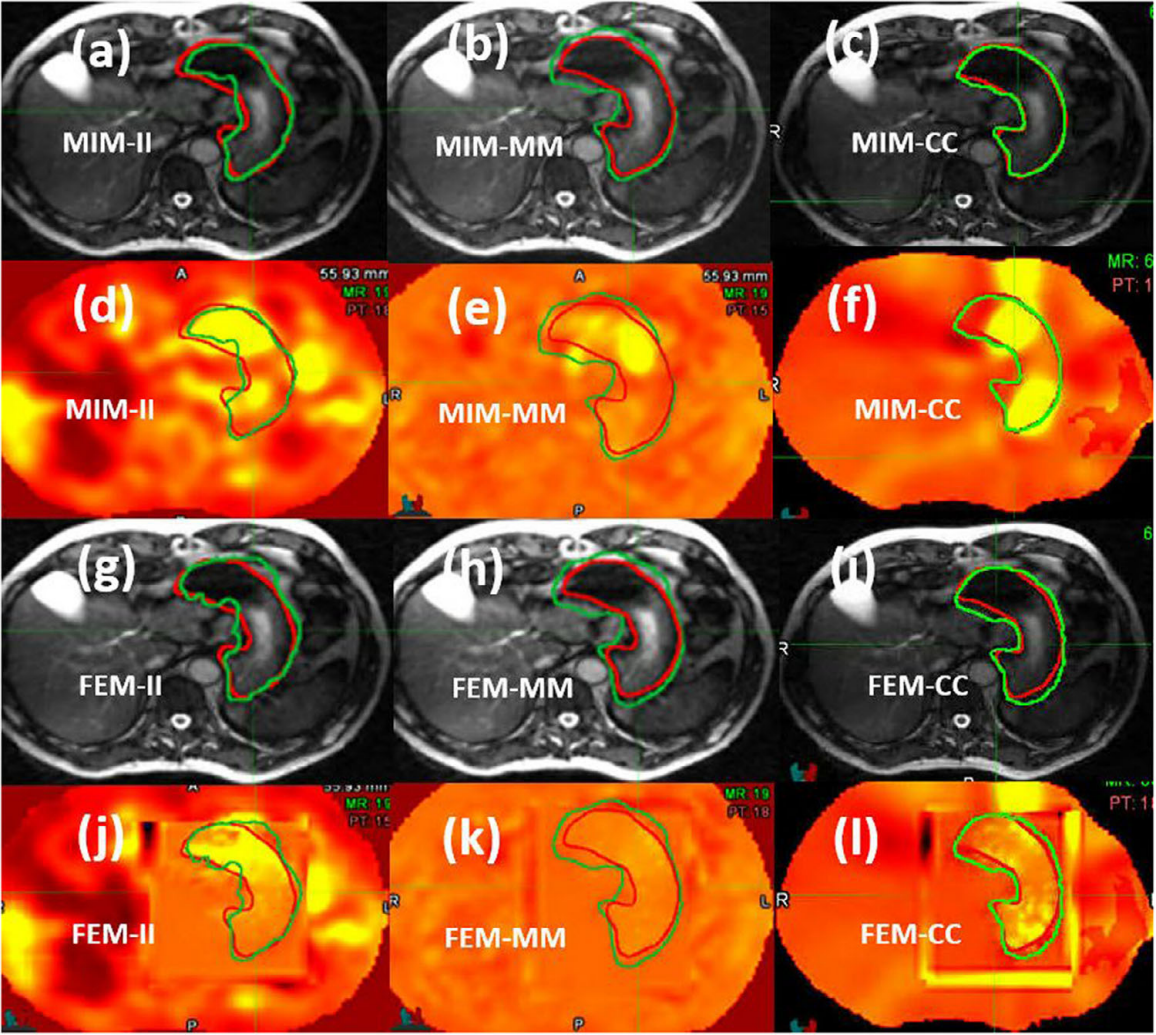
The MIM registrations and their FEM modifications for patient 3: (a–c) the reference image and contour (red) overlaid by the mapped contour (green) for MIM‐II, MIM‐MM, and MIM‐CC registrations, respectively; (d–f) the JD distribution of MIM‐II, MIM‐MM, and MIM‐CC registrations; (g–i) the reference image and warped contours for FEM‐II, FEM‐MM, and FEM‐CC registrations; (j–l) the JD distribution of FEM‐II, FEM‐MM, and FEM‐CC registrations, respectively.

DIRs often exhibit significant errors within the stomach region. For patient 3, contour comparisons resulted in MDA values of 2.49, 2.83, and 0.80 mm for the MIM‐II, MIM‐MM, and MIM‐CC registrations, respectively, all within the 3 mm tolerance.^4^ However, the JD distributions of the resulting DVFs showed pronounced hot spots in the interior of the stomach, as illustrated in Figure 5d–f. STD‐JDs for the three registrations were 1.78, 0.63, and 1.22, respectively. Subsequent FEM‐based modifications led to minor alternations in the warped contours but significantly reduced these STD‐JDs to 0.53, 0.16, and 0.42, respectively. The warped contours and JD distributions for the FEM‐modified DVFs are presented in Figure 5g–i and Figure 5j–l, respectively.

The efficacy of the FEM modifications was evaluated using three metrics, and results were averaged over the 20 OARs. The mean and standard deviation for each metric are listed in Table 1. It can be found that the FEM‐based modification reduces STD‐JDs by 60.6%, 57.3%, and 52.7% for MIM‐II, MIM‐MM, and MIM‐CC algorithms, respectively. One‐tailed WMW tests were performed between the STD‐JDs of the MIM registrations and their FEM modifications, resulting in *p*‐value of 0.0035 for MIM‐II, 0.0002 for MIM‐MM, and 0.0084 for MIM‐CC, all smaller than the significances level of 0.05. In contrast, the MIM and FEM registrations do not have any significant difference in terms of DSC and MDA as shown in Table 1.

**TABLE 1:** MIM and FEM registrations evaluated over a total of 20 OARs from 10 patients.

DIR Methods	DSC		MDA		STD‐JD	
	Mean ± SD	*p*‐values	Mean ± SD	*p*‐values	Mean ± SD	*p*‐values
MIM‐II	0.77 ± 0.15	0.933	3.07 ± 2.42	0.970	0.44 ± 0.39	0.0035
FEM‐II	0.77 ± 0.15		3.02 ± 2.35		0.17 ± 0.12	
MIM‐MM	0.80 ± 0.12	0.986	2.39 ± 1.30	0.976	0.24 ± 0.14	0.0002
FEM‐MM	0.80 ± 0.12		2.35 ± 1.27		0.10 ± 0.04	
MIM‐CC	0.94 ± 0.03	0.870	0.62 ± 0.27	0.951	0.33 ± 0.29	0.0084
FEM‐CC	0.93 ± 0.04		0.65 ± 0.31		0.15 ± 0.12	
CM‐FEM	0.90 ± 0.11	NA	1.04 ± 0.72	NA	0.21 ± 0.09	NA

Note that the boundary condition of a hybrid FEM registration is determined by displacements obtained from a preliminary registration. If the preliminary registration has significant errors at organ boundaries, these errors could be propagated into the boundary condition, thereby diminishing the effectiveness of the hybrid FEM registration. However, as demonstrated in Table 1, FEM modifications substantially reduce the variation of Jacobian determinants of the MIM registrations in interior regions without compromising their performance at organ boundaries.

### 3.3 Application of the hybrid FEM algorithm

**FIGURE 6:**
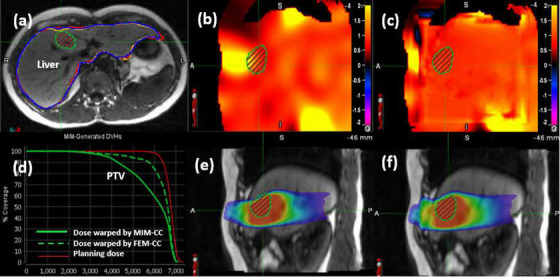
Reference image for patient 4: (a) comparison of the warped liver contours by MIM‐CC (yellow) and FEM‐CC (blue) with the target contour (red); (b) and (c) sagittal cuts of the JD maps for MIM‐CC and FEM‐CC, respectively; (d) DVHs of the PTV for the planning dose and MIM‐CC and FEM‐CC warped doses; (e) and (f) sagittal cuts of the MIM‐CC and FEM‐CC warped doses.

Dose mapping was performed for a patient with a planning target volume (PTV) of 19.8 cc located within the liver. DIR was performed from the second fraction to the first fraction using MIM‐CC and the resultant DVF was modified with FEM‐CC. The liver contours warped by MIM‐CC (yellow) and FEM‐CC (blue) were compared to the reference contour (red) as shown in Figure 6a. JD maps calculated for MIM‐CC and FEM‐CC are shown in Figure 6b,c. It can be found that the FEM‐CC modification has removed the hot spot near the PTV contour, with STD‐JDs reduced from 0.48 to 0.05 on average in the liver.

The DVHs of the planning dose and the warped doses by MIM‐CC and FEM‐CC were shown in Figure 6d. It can be observed that the FEM‐CC modification leads to an increase in V_60_ from 59.2% to 84.1% for the PTV and from 7.02% to 7.99% for the liver.

## 4 DISCUSSION

The MIM software version 7.06 includes three image or contour‐based DIR algorithms as documented in.^11^ While these algorithms have shown success in registering images such as cervical CTs,^24^ thoracic 4DCTs or CTs,^21^, ^25^ and head and neck MRIs,^26^ their performance in registering abdominal MRIs has not been comprehensively evaluated.^27^ In this study, we compared these algorithms using metrics recommended by the AAPM task group and other researchers and evaluated their performance at organ boundaries and interior regions, respectively. To address large uncertainties identified in interior regions, we developed a mechanical modeling method to adjust resultant DVFs to facilitate uninterrupted clinical procedures. Unlike image‐based registration algorithms susceptible to changes in image characteristics, such as imaging artifacts and low image contrasts,^9^ mechanical modeling operates independently of these image characteristic.^10^ When a MIM registration is evaluated to be accurate at organ boundaries, its boundary displacements can be directly utilized in the hybrid FEM technique to reduce uncertainties in the interior region of these organs (Figure 4). This approach is different from other FEM registration methods that require additional derivation and validation of boundary conditions.^10^

The three DIR algorithms MIM‐II, MIM‐MM, MIM‐CC demonstrate an increased order of performance at organ boundaries, as measured with the metrics of DSC and MDA. For the MIM‐II algorithm, the DSC in Table 1 is 0.77 ± 0.15 for abdominal MRIs, which closely aligns with the DSC values of 0.8 ± 0.1 for lung CT images^25^ and 0.76 ± 0.15 for pelvis CT images.^24^ Furthermore, while DSC‐based assessments are structure‐size‐dependent and could be influenced by the type of evaluated ORAs,^20^ the evaluations performed using the two metrics are shown to be consistent in this study. In interior regions, MIM‐MM shows the best performance, followed by MIM‐CC and then MIM‐II. Unlike image‐based DIRs that could have large uncertainties in low‐contrast regions,^10^ the hybrid FEM technique shows its capability to reduce DIR uncertainties in these regions (Figure 4). When contours are delineated on both treatment and reference images, DIR can be performed with the CM‐FEM method. While MIM‐CC shows superior performance to CM‐FEM in terms of DSC and MDA, it produces noticeable hot and cold spots in its JD maps. The suboptimal performance of CM‐FEM at organ boundaries could be due to multiple factors, such as the search range for matching contour point pairs and displacement transferring between boundary voxels and tetrahedral nodes. Tuning these parameters might help improve CM‐FEM's performance.

It is important to note that certain applications, such as “Reg Refine” in MIM, permit users to update a deformation registration through manual alignments.^11^ However, performing such alignments is neither straightforward in low‐contrast regions nor efficient for online applications, and the results could be operator‐dependent. A recent study revealed that, with CT images from 35 head and neck cancer patients, the “Reg Refine” registrations conducted by experienced operators were classified as good, fair and bad, corresponding to STD‐JD values of 0.05, 0.21, and 0.68, respectively.^28^ In contrast, the hybrid FEM method automatically updates the MIM registrations in interior regions, reducing STD‐JDs from 0.44 to 0.17 for MIM‐II, from 0.33 to 0.15 for MIM‐CC, and from 0.24 to 0.10 for MIM‐MM. The one‐tailed Wilcoxon‐Mann‐Whitney tests show that these improvements are statistically significant, with all *p*‐values less than 0.0084. Although the MIM and FEM registrations exhibit negligible differences at organ boundaries (Table 1), reducing DIR uncertainties in interior regions is crucial for accurate dose accumulation in these organs.

In this study, we evaluated three MIM registration methods and developed a hybrid FEM method to mitigate uncertainties of the MIM registrations within interior regions. The implications of these enhancements on dose deformation may vary depending on several factors, including dose gradient, JD distribution, OAR volume, and evaluation baseline and criterion. For example, compared to the liver in Figure 6a, the PTV is a small target located in a high dose‐gradient region, and is therefore more sensitive to the FEM modification. Since there is no ground truth DVF available, the dosimetry enhancements resulting from the FEM modification cannot be quantified in this study. Nevertheless, judging from the accurately mapped contours and reduced JD uncertainties in the interior region of the liver, FEM‐CC appears to offer a more dependable DVF for subsequent dose mappings compared with MIM‐CC. In future studies, we will systematically consider these factors to thoroughly investigate the dosimetric consequences of DIR uncertainties and their FEM corrections across various treatment sites and clinical scenarios. Furthermore, given that the current FEM program communicates with the MIM software via DICOM import and export, necessitating additional time for data transfer and processing (Figure 1), we will explore more efficient methods to seamlessly integrate this program with the MIM software.

## 5 CONCLUSION

Among the three MIM DIR algorithms, MIM‐CC shows the smallest errors in terms of MDA and DSC but exhibits significant Jacobian uncertainties in the interior regions of abdominal organs. The hybrid FEM technique effectively mitigates the Jacobian uncertainties in these regions.

## AUTHOR CONTRIBUTIONS

The study was conceived and designed collaboratively by all three authors. Zhong H contributed to software development and evaluation. Kainz K contributed to literature review and data collection. Paulson E provided expertise in MRI techniques and their applications communicated in the manuscript.

## CONFLICT OF INTEREST STATEMENT

The authors declare no conflicts of interest.

## Supporting information

Supporting Information

## Data Availability

We acknowledge any interest in accessing our code, solutions, or data for replication and further research purposes. However, due to constraints such as the extensive size and proprietary nature of our code and data, we may not be able to share it in its entirety. We are committed to promoting transparency and reproducibility and are available to provide relevant portions of the code, specific algorithms, methodologies, or data upon request. Please contact the corresponding author for inquiries regarding access to our work.
