# Supplementary material for: Evaluation and mitigation of deformable image registration uncertainties for MRI‐guided adaptive radiotherapy
Source: J Appl Clin Med Phys. 2024 Apr 18;25(6):e14358. doi: 10.1002/acm2.14358 (PMC11163488; doi:10.1002/acm2.14358)
Supplement: Supplementary file 1 — Supporting Information [file ACM2-25-e14358-s001.docx]

Evaluation and mitigation of deformable image registration uncertainties for MRI-guided adaptive radiotherapy: supplementary material

Hualiang Zhong, Kristofer K Kainz, Eric S Paulson

Department of Radiation Oncology

Medical College of Wisconsin, Milwaukee, WI, USA

1. **Evaluation of DIR algorithms**

In this study, we evaluated four DIR algorithms: MIM-II, MIM-MM and MIM-CC in MIM version 7.06 (MIM Software Inc, Cleveland, OH), and CM-FEM in an in-house developed FEM program. These algorithms were applied to MR images acquired from 10 MR-Linac patients. Figure 1 shows MR images acquired from an MR-Linac patient at the first and second fractions, where the contents of the small bowel show different image intensities. When MIM-II was used to register the two images, the varying image intensities in the small bowel compromised the performance of the MIM-II registration on the left kidney, resulting in the warped contour (yellow) deviating significantly from the target contour (pink) in Figure 1(c).


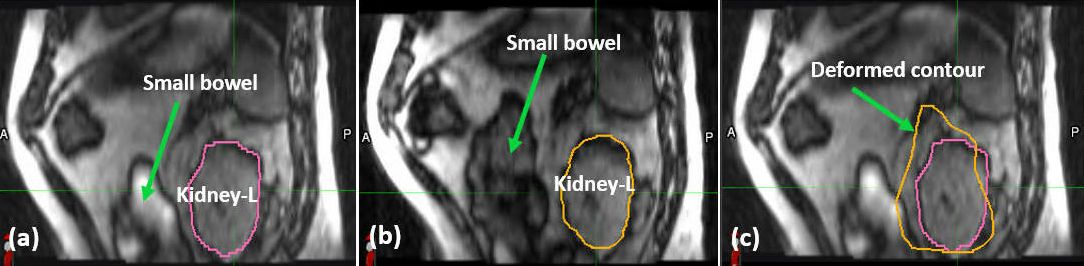


Figure 1. Daily MR images from an MR-Linac patient: (a) the left kidney contoured on the target image (fraction 1); (b) the left kidney contoured on the source image (fraction 2); (c) the MIM-II warped contour and its target contour for the left kidney overlaid on the target image.

1. **Evaluation of the hybrid FEM algorithm**

A hybrid registration method was developed that uses a mechanical modeling technique to improve the performance of MIM registrations within individual organs. DVFs from the MIM and FEM registrations were evaluated across 20 organs at risk (OARs) that are close to treatment targets. These OARs include the liver and stomach from 4 patients, duodenum from 3 patients, and right kidney and pancreas from 2 patients. Additionally, the left kidney, spleen, gross tumor volume, spinal cord and portal vein were evaluated from 4 patients each. The evaluation metrics, including DICE similarity coefficient (DSC), mean displacement to agreement (MDA) and standard deviation of the Jacobian determinant (STD-JD), were calculated on each of these OARs, and boxplots for these calculated metrics were shown in Figures 2-4.


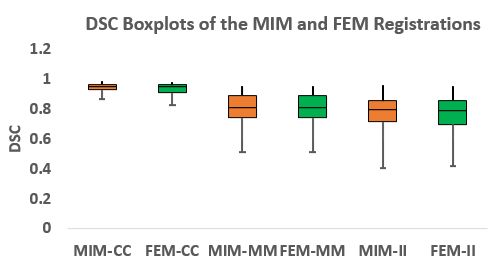


Figure 2. Boxplots illustrating the DSC of the MIM and FEM registrations calculated across 20 organs at risk. A DSC=1 indicates the best performance of a registration in contour comparisons.


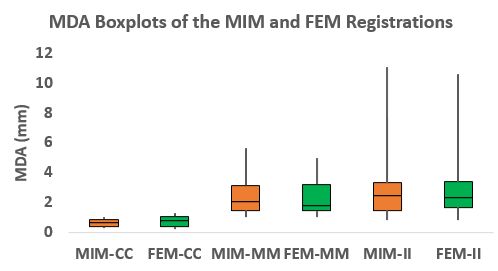


Figure 3. Boxplots illustrating the MDA of the MIM and FEM registrations calculated across 20 organs at risk. An MDA=0 indicates the best performance of a registration at organ boundaries.


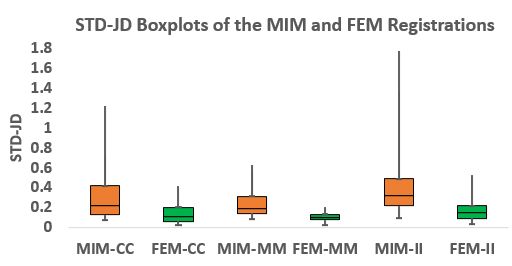


Figure 4. Boxplots illustrating the STD-JD of the MIM and FEM registrations calculated across 20 organs at risk. An STD-JD=0 indicates the best quality of a registration in interior regions.
